# Endometrial preparation for embryo transfer: reproductive outcomes of modified natural cycle versus hormone replacement therapy

**DOI:** 10.61622/rbgo/2026rbgo66

**Published:** 2026-10-30

**Authors:** Laura Caetano de Sá, Luma Soares Fagundes, Rivia Mara Lamaita, Ana Márcia de Miranda Cota

**Affiliations:** 1 Faculdade Ciências Médicas de Minas Gerais Belo Horizonte MG Brazil Faculdade Ciências Médicas de Minas Gerais, Belo Horizonte, MG, Brazil.; 2 Centro de Reprodução Humana Rede MaterDei de Saúde Belo Horizonte MG Brazil Rede MaterDei de Saúde, Centro de Reprodução Humana, Belo Horizonte, MG, Brazil.

**Keywords:** Embryo transfer, Infertility, Anovulation, Endometriosis, Reproductive techniques, Pregnancy rate, Hormone replacement therapy

## Abstract

**Objective:**

Compare reproductive outcomes between modified natural cycle (mNC) and hormone replacement therapy (HRT) for endometrial preparation in embryo transfer.

**Methods:**

Retrospective cohort study (2020–2024) including 180 cycles that reached embryo transfer at a tertiary center. Primary outcome was biochemical pregnancy (β-hCG positivity), and clinical pregnancy was secondary. Multivariable logistic regression adjusted for baseline imbalances (age, infertility duration, endometriosis, unexplained infertility) and embryonic factors (developmental stage, PGT-A status) was performed. Sensitivity analyses assessed embryonic heterogeneity.

**Results:**

Cohort comprised 68.9% HRT and 31.1% mNC cycles. Baseline differences were observed: mNC had higher rates of endometriosis (41% vs. 25%; p = 0.029), unexplained infertility (30% vs. 9.7%; p < 0.001), and longer infertility duration (median 5 vs. 3 years; p = 0.003), while anovulation was more frequent in HRT (22% vs. 5.4%; p = 0.006). No statistically significant difference in biochemical pregnancy rates was observed between groups (OR 0.87, 95% CI 0.45–1.65; p = 0.682). After adjustment, endometrial preparation protocol was not independently associated with biochemical pregnancy (aOR 1.31; 95% CI 0.51–3.35; p = 0.568). Sensitivity analyses showed consistent results despite blastocyst transfer predominance (75%).

**Conclusion:**

After adjustment for clinical and embryonic confounders, no statistically significant association was observed between endometrial preparation protocol and β-hCG levels or clinical pregnancy among cycles that achieved embryo transfer. Despite retrospective design, limited progesterone data, and exclusion of canceled cycles, mNC may represent a feasible physiological alternative in selected ovulatory patients, although prospective studies are needed.

## Introduction

The aging of the Brazilian population, combined with contemporary cultural influences on fertility, has increased the relevance of assisted reproductive technologies (ART) in the country. In 2019, 99,112 embryos were cryopreserved and 25,959 embryo transfers were performed,^(1)^ highlighting the impact of reproductive medicine on future generations and emphasizing the importance of high-quality fertility treatments.

In frozen-thawed embryo transfer cycles, proper endometrial preparation is critical for treatment success, as it aims to synchronize endometrial and embryonic development. Various approaches exist, including natural cycle (NC), modified natural cycle (mNC), stimulated cycle (SC), and hormone replacement therapy (HRT), but no consensus has been reached regarding the optimal protocol. While SC is often reserved for anovulatory patients, mNC and HRT are more commonly used and are the focus of this study.

In NC, embryo transfer is synchronized with the luteal phase, taking advantage of the patient's spontaneous ovulation and relying on endogenous estrogen and progesterone for endometrial proliferation and stabilization.^(2)^ The procedure requires repeated hormone measurements and transvaginal ultrasounds to monitor follicular growth, ovulation, and endometrial characteristics. In mNC, ovulation is triggered with human chorionic gonadotropin (hCG), allowing programmed embryo transfer.^(3)^

Although NC represents the most physiological approach, its clinical application may be limited by the need for intensive monitoring and the unpredictability of spontaneous ovulation, which can complicate cycle scheduling and increase cancellation rates. In contrast, mNC, through the use of hCG to trigger ovulation, allows for greater cycle control and flexibility while preserving corpus luteum function and a more physiological hormonal environment. For these reasons, mNC has been increasingly adopted in clinical practice and represents a pragmatic and clinically relevant alternative to NC.

HRT protocols, in contrast, induce hormonal changes through exogenous administration, offering greater flexibility and lower cycle cancellation rates, although they may disrupt female homeostasis due to medication side effects. The choice of protocol should be individualized, considering ovulatory status, ovarian response, infertility etiology, and other patient-specific factors.^(4)^

In addition, SC protocols - typically used in anovulatory patients - combine exogenous gonadotropin stimulation with cycle monitoring and may serve as an alternative to HRT. However, SC is less commonly applied in routine practice due to higher costs and more intensive monitoring requirements.

Current evidence comparing endometrial preparation strategies in frozen–thawed embryo transfer cycles remains heterogeneous and, in many cases, inconclusive.^(3,4)^ Some observational studies and secondary analyses have suggested that NC and mNC protocols may be associated with favorable obstetric outcomes, including reduced risks of hypertensive disorders and placental complications.^(4,5)^ However, these findings are not consistent across the literature and are largely derived from non-randomized data, limiting causal inference.

In contrast, HRT cycles, which are characterized by the absence of a corpus luteum, have been associated in some studies with increased risks of adverse maternal and perinatal outcomes, such as preeclampsia, gestational hypertension, abnormal placentation, large-for-gestational-age infants, postpartum hemorrhage, and preterm birth.^(6–10)^ Nonetheless, the underlying mechanisms remain under investigation, and the extent to which these risks are attributable to the endometrial preparation protocol itself is still debated.

From a physiological perspective, the presence of a corpus luteum in NC and mNC cycles may contribute to a more natural hormonal profile, potentially supporting endometrial receptivity and early placentation.^(8)^ However, other studies have reported no significant differences in reproductive outcomes between NC and HRT protocols in ovulatory women.^(3)^

Given these considerations, this study aims to analyze reproductive outcomes according to the method of endometrial preparation in frozen-thawed embryo transfer cycles, comparing mNC versus HRT.

## Methods

### Study design and population

This retrospective cohort study was conducted in the Human Reproduction Department of a hospital in Minas Gerais. We included all patients who underwent frozen embryo transfer (FET) with either a modified natural cycle (mNC) or hormone replacement therapy (HRT) protocol between 2020 and 2024. The cohort was defined exclusively by patients who reached embryo transfer, and the analysis was performed retrospectively from that point. Because our starting point was the embryo transfer itself, patients who initiated preparation but did not reach transfer (cycle cancellations) were not part of the dataset and could not be analyzed. This methodological choice allowed for a homogenous cohort of completed FET cycles but limited the ability to compare cancellation rates between protocols. Inclusion criteria encompassed women of all ages diagnosed with infertility, who underwent frozen embryo transfer cycles. Patients were eligible regardless of race, ethnicity, skin color, sexual orientation, or socioeconomic status. Exclusion criteria included patients with incomplete medical records.

### Endometrial preparation protocols

Modified natural cycle: Patients with regular ovulatory cycles were monitored using serial transvaginal ultrasounds starting in the early follicular phase and repeated every 2–3 days from cycle day 10 to monitor follicular growth. When the dominant follicle diameter reached 18mm, injection of r-HCG (recombinant human chorionic gonadotropin) 250mcg was performed to trigger ovulation. Due to the administration of the r-HCG to trigger ovulation, the endometrial preparation was considered modified (mNC). Luteal support began 2 days after r-HCG administration and was administered as vaginal micronized progesterone (P) 800mg per day (400mg twice daily). Embryo transfer was scheduled based on the embryo stage: cleavage stage or blastocyst. Cleavage stage embryos were transferred on the 5th day after injection of hCG (P+3) and blastocyst on the 7th day after injection of hCG (P+5) (Figure 1). Progesterone levels were assessed when appropriate, on the day of r-hCG injection and on the day of embryo transfer. Micronized progesterone use was continued until 12th gestational week in case of pregnancy.

**Figure 1:**
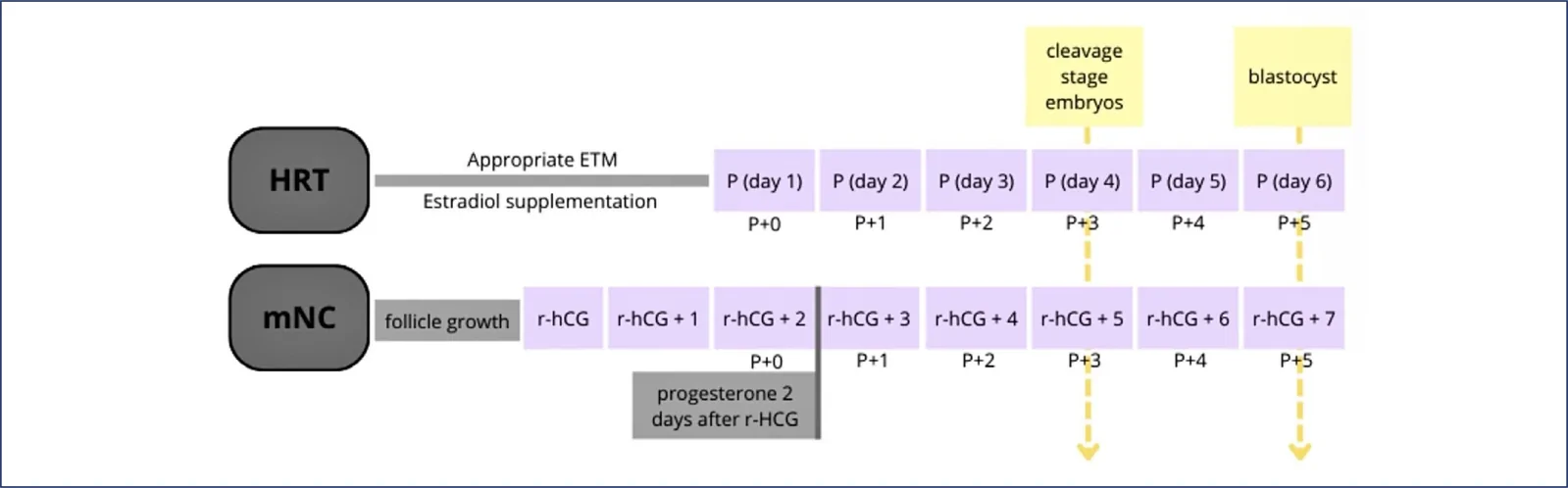
Endometrial preparation protocol for frozen embryo transfer uses at MaterDei Hospital. The flor Heart was created by the authors based on the instituion's clinical protocol.

HRT: Hormone Replacement Therapy; mNC: modified natural cycle; ETM: endometrial thickness; P: progesterone; r-HCG: recombinant hormone chorionic gonadotropin

### Hormone replacement therapy

Endometrial preparation was achieved using exogenous estradiol, orally, as a fixed dose of 6 mg per day administered from day 2 or 3 of the menstrual cycle followed by the first ultrasound monitoring. Usually, after 10-14 days of estradiol administration, ultrasound examination was performed for endometrial thickness measurement. Upon achieving a minimum endometrial thickness of ≥7 mm, progesterone supplementation (vaginal micronized progesterone 400mg twice daily, a total of 800 mg per day) was initiated, and embryo transfer was scheduled accordingly, matching the embryo development stage. Cleavage stage embryos were transferred on the 4th day (P+3) and blastocyst on the 6th day after progesterone administration (P+5) (Figure 1). Progesterone levels were assessed when appropriate, on the day of starting progesterone use and on the day of embryo transfer, as part of routine clinical practice. The timing of sample collection was standardized according to institutional protocol, and values were interpreted within the clinical context of each endometrial preparation strategy. Estradiol and micronized progesterone were continued until 12th gestational week in case of pregnancy. In both endometrial preparations, mNC and HRT, vaginal micronized progesterone (P) 800mg per day (400mg twice daily) was administered for luteal phase support until 12th gestational.

### Embryo transfer and outcome measures

All embryo transfers were performed under ultrasound guidance using a soft catheter by experienced reproductive specialists. Transfers were performed by multiple clinicians. To reduce the risk of bias, all procedures followed standardized strict institutional protocol (regarding catheter choice, placement, and transfer technique) and were conducted in a single operating room under identical environmental conditions. Single and double blastocyst-stage embryos were transferred, according to patient wishes and age. Luteal phase support was continued until 12 weeks of gestation in confirmed pregnancies. The primary outcome was biochemical pregnancy rate (defined by beta-hCG positivity) Secondary outcomes included clinical pregnancy rate (defined as visualization of a gestational sac with fetal heartbeat at 6–7 weeks), miscarriage rate and ectopic pregnancy rate. The characteristics analyzed from each cycle were: age at the time of oocyte retrieval; duration of infertility; and the presence of specific infertility-related factors such as genetic disorders, endometriosis, tubal factors, uterine fibroids, chronic anovulation, unexplained infertility and male factor infertility. Additional variables included endometrial thickness on the day of embryo transfer, serum progesterone levels on the day of transfer, embryo development stage (cleavage-stage or blastocyst), the number of embryos transferred in the cycle, and whether preimplantation genetic testing (PGT) was performed.

### Statistical analysis

Unadjusted comparative analyses were performed to assess baseline characteristics and reproductive outcomes between groups, aiming to evaluate group comparability and identify potential confounders. Continuous variables were expressed as median (interquartile range) and compared using the Mann–Whitney U test. Categorical variables were expressed as absolute and relative frequencies and compared using the Chi-square test or Fisher's exact test, as appropriate.

### Ethical aspects

This is a retrospective study involving human subjects, conducted in accordance with the ethical principles outlined in the Declaration of Helsinki and the guidelines established by Resolution No. 466/2012 (Brazilian Ministry of Health).^(11)^ The research protocol was submitted to and approved by Research Ethics Committee of Mater Dei Health Network under CAAE 82748624.9.0000.5128 and approval opinion number 7.042.050. Given the retrospective nature of the study, the requirement for individual informed consent was waived. All information obtained was treated with strict confidentiality, in compliance with the Researcher's Commitment Agreement and Data Use Commitment Agreement (TCUD).

## Results

### Clinical characteristics of the study participants

A total of 185 endometrial preparation cycles for frozen-thawed embryo transfer were included in the database. Five cycles were excluded from the primary analysis due to extreme maternal age, aiming to reduce heterogeneity. Of the 180 cycles remaining, 68.9% were HRT cycles (n = 124) and 31.1% were mNC cycles (n = 56). The mean age of the patients at the time of oocyte retrieval was 38 years, ranging from 35 to 44 years, with no statistically significant differences in age between the two groups (p = 0.4). Likewise, endometrial thickness showed no statistically significant difference (median 8.55 mm in the HRT group vs. 8.00 mm in the mNC group; p = 0.4).

The modified natural cycle group had significantly more patients with endometriosis (p = 0.029), unexplained infertility (p < 0.001), and a longer duration of infertility, with a median of 3 years in the HRT cycle group and 5 years in the mNC group (Q1-Q3: 2-5 and 3-6 years, respectively; p = 0.003). The HRT group had more patients with anovulation (p = 0.006), as expected, since this distribution is consistent with current clinical practice, where artificial cycles are strategically employed to overcome the unpredictability of follicular growth in anovulatory patients, ensuring adequate endometrial preparation regardless of endogenous ovarian activity. There were no significant differences observed for genetic disorders (p = 0.059), tubal factor infertility (p = 0.076), uterine myomatosis (p = 0.3), or male factor infertility (p = 0.2). Detailed baseline characteristics and unadjusted comparisons are presented in tables 1 and 2.

**Table 1:** Baseline characteristics

Characteristics		Total* n=180 n(%)	Endometrial preparation		p-value**
			Hormone Replacement Therapy Cycle* n=124 n(%)	Modified Natural Cycle* n= 56 n(%)	
Age at oocyte retrieval (years)		38.0 (35.0, 40.0)	38.0 (35.0, 40.0)	38.0 (36.0, 40.0)	0.4
Endometrial thickness (mm)		8.10 (7.50, 10.00)	8.55 (7.50, 10.00)	8.00 (7.30, 10.00)	0.4
Infertility duration (years)		3.0 (2.0, 6.0)	3.0 (2.0, 5.0)	5.0 (3.0, 6.0)	0.003
Embryonic Stage (n=179)					0.4
Blastocyst		134(75)	90(73)	44(80)	
Embryo in cleavage		45(25)	34(27)	11(20)	
Number of embryos transfer					0.6
	1	79(44)	53(43)	26(46)	
	2	99(55)	70(56)	29(52)	
	3	2(1.1)	1(0.8)	1(1.8)	
PGT-A					0.13
	No	161(89)	108(87)	53(95)	
	Yes	19(11)	16(13)	3(5.4)	
Infertility factors					
Anovulation					0.006
	No	150(83)	97(78)	53(95)	
	Yes	30(17)	27(22)	3(5.4)	
Genetic disease					0.059
	No	171(95)	115(93)	56(100)	
	Yes	9(5.0)	9(7.3)	0(0)	
Endometriosis					0.029
	No	126(70)	93(75)	33(59)	
	Yes	54(30)	31(25)	23 (41%)	
Tubal factor					0.076
	No	159(88)	106(85)	53(95)	
	Yes	21(12)	18(15)	3(5.4)	
Advanced maternal age					0.5
	No	87(48)	62(50)	25(45)	
	Yes	93(52)	62(50)	31(55)	
Uterine myomatosis					0.3
	No	163(91)	114(92)	49(88)	
	Yes	17(9.4)	10(8.1)	7(13)	
Unexplained infertility					<0.001
	No	151(84)	112(90)	39(70)	
	Yes	29(16)	12(9.7)	17(30)	
Male factor					0.2
	No	95(53)	61(49)	34(61)	
	Yes	85(47)	63(51)	22(39)	

* Median (Q1, Q3);

** Wilcoxon rank-sum test; Chi-square test of Independence; Fisher's exact test

**Table 2:** Unadjusted comparison of reproductive outcomes between mNC and HRT Groups

Characteristics		Total N = 180 n(%)	Endometrial preparation N(%)		p-value*
			Hormone Replacement Therapy Cycle N = 124 n(%)	Modified Natural Cycle N = 56 n(%)	
Beta-hcg test					0.8
	Negative	110(61)	75(60)	35(63)	
	Positive	70(39)	49(40)	21(38)	
Number of gestational sacs					>0.9
	0	111(62)	76(61)	35(63)	
	1	60(33)	41(33)	19(34)	
	2	9(5.0)	7(5.6)	2(3.6)	
Did it progress to clinical pregnancy?					>0.9
	No	118(66)	81(65)	37(66)	
	Yes	62(34)	43(35)	19(34)	
Other outcomes (n=8)					0.8
	Miscarriage	6(3.3)	5(4.0)	1(1.8)	
	Ectopic pregnancy	2(1.1)	1(0.8)	1(1.8)	

* Wilcoxon rank-sum test; Chi-square test of Independence; Fisher's exact test

### Reprodutive outcomes

The overall β-hCG positivity rate was 40% in the HRT group and 38% in the mNC group, with no statistically significant difference (p = 0.8) (Table 2). Overall, 39% of cycles (70/180) resulted in laboratory-confirmed pregnancy, of which 62 progressed to clinical pregnancy, 6 resulted in miscarriage, and 2 were ectopic pregnancies (Table 2). No statistically significant differences were observed between groups in the number of gestational sacs (p>0.9) or in adverse outcomes, including miscarriage and ectopic pregnancy.

### Factors associated with biochemical pregnancy

When evaluating the primary reproductive outcome, the crude analysis showed no significant difference between the HRT and mNC groups regarding biochemical pregnancy rates (crude OR 0.87, 95% CI 0.45–1.65; p = 0.682). After adjusting for potential confounding factors — including the higher prevalence of endometriosis, unexplained infertility, embryo stage, and PGT-A — multivariable logistic regression confirmed that endometrial preparation protocol was not an independent predictor of biochemical pregnancy (adjusted OR 1.31, 95% CI 0.51–3.35; p = 0.568). Among the covariates, only maternal age was significantly associated with the outcome (adjusted OR 0.86, 95% CI 0.76–0.96; p = 0.011), indicating a decrease in the odds of biochemical pregnancy with increasing age. No significant associations were observed for the remaining variables.

### Logistic regression analysis

To account for potential confounding, logistic regression models were constructed. A crude model evaluated the unadjusted association between endometrial preparation protocol and biochemical pregnancy (Table 3). A multivariable model adjusted for clinically relevant covariates, including age at oocyte retrieval, infertility duration, endometriosis, unexplained infertility, embryo stage, and PGT-A (Adjusted Analysis) (Table 4). Results are presented as odds ratios (OR) and adjusted odds ratios (aOR) with 95% confidence intervals.

**Table 3:** Unadjusted logistic regression analysis of the association between endometrial preparation and positive β-hCG

Variable	Unadjusted OR	95% CI	p-value
Endometrial preparation: modified natural cycle	0.87	0.45 - 1.65	0.682

OR: Odds Ratio; CI: Confidence Interval. Crude logistic regression model including only endometrial preparation protocol as predictor. Outcome: positive β-hCG. Reference category: HRT. A p-value < 0.05 was considered statistically significant

**Table 4:** Multivariable logistic regression analysis for factors associated with positive beta-hCG

Variables	Adjusted OR	95% CI	p-value
Endometrial preparation: Modified Natural Cycle	1.31	0.51 - 3.35	0.568
Age at oocyte retrieval	0.86	0.76 - 0.96	0.011
Infertility duration	1.05	0.9 - 1.22	0.531
Endometriosis	0.49	0.18 - 1.29	0.156
Unexplained infertility	0.67	0.22 - 2	0.475
Embryonic stage: Cleavage	0.48	0.17 - 1.3	0.162
PGT-A	1.52	0.42 - 5.67	0.522

OR: Odds Ratio; CI: Confidence Interval. Multivariable logistic regression model including endometrial preparation (modified natural cycle vs. HRT), age at oocyte retrieval, infertility duration, endometriosis, unexplained infertility, embryonic stage (cleavage vs. blastocyst), and PGT-A. Outcome: positive β-hCG. Reference category: HRT for endometrial preparation and blastocyst for embryonic stage. A p-value < 0.05 was considered statistically significant

The robustness of the multivariate model was validated through multicollinearity diagnostics. We calculated the Variance Inflation Factor (VIF) for all independent variables to rule out redundant correlations that could bias the coefficients. All covariates exhibited VIF values < 1.5, indicating the absence of multicollinearity and ensuring the stability of the aOR estimates (Supplementary Material - Table 1S).

### Embryo developmental stage and sensitivity analysis

Regarding embryo developmental stage, blastocyst transfers accounted for the majority of cycles (75%), while cleavage-stage embryos represented 25% of the sample (Table 1). The proportion of blastocyst transfers was 80% in the mNC group compared to 73% in the HRT group, a difference that was not statistically significant (p = 0.4; Table 1). Furthermore, the number of embryos transferred per cycle did not differ significantly between groups, with the majority receiving one or two embryos (p = 0.6; Table 1). Importantly, no statistically significant differences were observed regarding PGT-A testing (13% in HRT vs. 5.4% in mNC, p = 0.13) (Table 1).

Sensitivity analyses were subsequently performed including all 185 cycles to evaluate the robustness of the results, by stratifying embryo developmental stage (cleavage-stage vs. blastocyst). Sensitivity analyses were performed to address sample heterogeneity by stratifying the dataset into more homogeneous subgroups, particularly according to embryo developmental stage (blastocyst vs other cleavage states). These analyses were conducted using the full dataset (n = 185), prior to exclusion of cycles with extreme maternal age, and are presented in Table 5 and Supplementary Material - Table 2S.

**Table 5:** Distribution of clinical characteristics and reproductive outcomes according to embryo development stage (Blastocyst vs Other Cleavage States)

Characteristic			Embryo development stage	
			Cleavage N= 46 n(%)	Blastocyst N = 139 n(%)
Endometrial preparation				
Hormone replacement therapy cycle			34(18.4)	93(50.3)
Modified natural cycle			12(6.5)	46(24.9)
Beta-hcg test				
	Negative		36(19.5)	78(42.2)
	Positive		10(5.4)	61(33)
Did it progress to clinical pregnancy?				
	Yes		10(5.4)	53(28.6)
	No		36(19.5)	86(46.5)
		No - miscarriage	0(0)	6(3.2)
		No - ectopic pregnancy	0(0)	2(1.1)

When evaluating reproductive outcomes, higher rates of β-hCG positivity and clinical pregnancy were observed in blastocyst transfers compared to cleavage-stage embryos, consistent with known differences in implantation potential (Table 5). The distribution of embryo stage between mNC and HRT cycles did not show a pattern suggestive of a major imbalance that could fully account for differences in outcomes (Tables 5 and Supplementary Material -Table 2S). The descriptive cross-tabulations (Tables 4 and Supplementary Material - Table 2S) created from the sensitivity analysis showed no major differences in the distribution of key clinical characteristics across the evaluated subgroups. These findings support the consistency of the primary results across different embryo stages and diagnostic profiles. These findings indicate the presence of embryo-related heterogeneity but suggest that this did not substantially influence the overall comparison between endometrial preparation protocols.

### Statistical analysis

Normality of continuous variables was assessed using the Shapiro–Wilk test. Detailed descriptive statistics and normality assessment results are presented in Supplementary Material - Table 3S. As most variables were not normally distributed, nonparametric methods were applied. A p-value < 0.05 was considered statistically significant. Analyses were performed using RStudio (version 4.5.0). Microsoft Excel was used to construct the database, and Microsoft Word was used for report editing. Data confidentiality was ensured through information coding and restricted access to files.

## Discussion

This study aimed to evaluate the effectiveness of modified natural cycle (mNC) versus hormone replacement therapy (HRT) endometrial preparation protocols in patients undergoing frozen-thawed embryo transfer in the context of in vitro fertilization. In the adjusted multivariate analysis, no significant differences were observed between the groups regarding reproductive outcomes, including biochemical pregnancy and clinical pregnancy rates. So, in this retrospective study, no statistically significant association was observed between the endometrial preparation protocol and the rates of positive β-hCG or clinical pregnancy among the cycles that achieved embryo transfer after adjustment for potential confounders.

These results are in line with growing evidence in the literature supporting the non-inferiority of modified natural cycle protocols in selected patient populations, especially those who are regularly ovulatory and present no contraindications to spontaneous cycle monitoring.^(2,3,12)^ Despite being a less interventionist approach, natural cycle preparation in our study was not associated with statistically significant differences in implantation or clinical pregnancy rates compared with HRT, even among patients with a longer history of infertility and a higher prevalence of endometriosis.

On the other hand, HRT protocols offer logistical advantages such as cycle predictability, reduced monitoring requirements, and better control over timing, which may benefit both patients and clinics^.(12)^. This flexibility is particularly important in patients with anovulation or irregular cycles, who represented a significantly larger proportion of the HRT group in our study. The use of estradiol orally allows for precise synchronization of endometrial development with embryo transfer, even in patients who do not ovulate naturally ^(4)^.

An important finding of our cohort was the baseline difference in infertility etiology between the groups. The mNC group had a significantly higher prevalence of endometriosis (41% vs. 25%, p = 0.029) and unexplained infertility, alongside a longer median duration of infertility. This likely reflects a selection bias inherent to clinical practice, where patients with regular ovulatory cycles—a prerequisite for mNC ^(4)^ may have a different etiological profile compared to those receiving HRT, who frequently present with ovulatory dysfunction (as evidenced by the higher anovulation rate in the HRT group). Interestingly, despite endometriosis being a known pro-inflammatory condition that could theoretically impair endometrial receptivity,^(13)^ no statistically significant differences in reproductive outcomes were observed between the protocols.

Exploring the study's inherent limitations, several factors should be considered when interpreting the findings. First, the retrospective and non-randomized design introduces the possibility of selection bias and residual confounding despite adjustment for relevant clinical and embryonic variables.^(14)^ As with any observational study, unmeasured or incompletely measured factors may have influenced the observed associations.

Selection bias must be acknowledged, as patients whose cycles were canceled before embryo transfer were excluded from the final analysis. Reasons for cancellation included patient withdrawal, loss to follow-up, impaired embryo development, incomplete medical records, and other clinical or logistical factors. Consequently, the present study reflects outcomes among completed embryo transfers rather than an intention-to-treat population, which may limit the generalizability of the findings and could have contributed to an overestimation of success rates.

Another important limitation relates to hormonal monitoring. Although progesterone levels represent a clinically relevant variable in endometrial preparation^(15,16)^, systematic measurements were unavailable for most cycles included in the study. The limited subset of available data showed substantial variability and lacked standardized timing of collection, precluding robust statistical analyses and the application of clinically meaningful thresholds. Consequently, progesterone levels were not incorporated into the primary analyses. Future prospective studies with standardized hormonal monitoring are needed to clarify their role in mNC and HRT cycles.

A further limitation concerns embryo-related heterogeneity. To preserve sample size and maintain real-world applicability, the cohort had to be structurally analyzed into two specific subgroups based on embryo developmental stage: blastocysts and embryos in cleavage (encompassing all non-blastocyst transfers). While this approach enhances external validity and better reflects routine clinical practice, it may also introduce additional sources of confounding, such as embryos with different developmental stages, variable embryo quality, and inconsistent use of preimplantation genetic testing for aneuploidy (PGT-A). ^(17,18)^ Therefore, residual confounding related to embryo characteristics cannot be completely excluded.

Another limitation concerns outcome assessment. Although pregnancy outcomes were evaluated, live birth rates – the gold-standard outcome in reproductive medicine^(19)^ could not be analyzed because the available sample size was insufficient to provide adequate statistical power. Therefore, the primary outcomes were restricted to β-hCG positivity and progression to clinical pregnancy. Additionally, obstetric outcomes, including hypertensive disorders of pregnancy, as well as patient-centered outcomes such as psychological burden, treatment costs, and patient satisfaction, were not assessed.

The study may have lacked sufficient statistical power to detect small but clinically meaningful differences between endometrial preparation protocols. As a single-center retrospective cohort, the sample size was limited, increasing the possibility of a Type II error. Consequently, the absence of statistically significant differences should be interpreted as a lack of evidence of a difference rather than evidence of equivalence between protocols. Larger prospective multicenter studies are needed to further evaluate these findings.

Finally, factors influencing the clinician's choice of endometrial preparation protocol, as well as other patient, laboratory, and embryo-related variables not captured in the database, may have contributed to residual confounding.

Despite these limitations, an important strength of the present study is its real-world design, reflecting routine IVF practice in a Latin American population that remains underrepresented in the literature.^(20)^ This characteristic may enhance the external validity of the findings and increase their applicability to similar clinical settings.

The modified natural protocol may be a suitable and effective option for selected patients, particularly those with regular ovulatory cycles, offering the advantage of a physiological environment and reduced medication exposure ^(4)^. Meanwhile, the hormone replacement therapy protocol remains a valuable strategy for patients requiring more predictable cycle control, especially in cases of anovulation or cycle irregularity. ^(12)^

Looking ahead, future research should aim to incorporate prospective randomized trials that not only compare clinical and biochemical pregnancy rates, but also evaluate live birth, perinatal outcomes, and maternal health. Integrating patient-centered outcomes such as treatment satisfaction, psychological well-being, and economic costs will help define the true value of each protocol in real-world practice. Moreover, exploring the molecular and immunological mechanisms underlying endometrial receptivity in natural versus artificial cycles may provide new insights for individualized reproductive medicine.

## Conclusion

Our findings did not identify statistically significant differences in positive β-hCG or clinical pregnancy rate between modified natural cycle and hormone replacement therapy endometrial preparation protocols in frozen-thawed embryo transfer cycles. Clinical pregnancy, biochemical pregnancy, and gestational sac development did not differ significantly between groups. These findings remained consistent after adjustment for potential confounding factors. Given the absence of significant differences in reproductive outcomes, the choice of protocol should be based on individual clinical profiles, patient preferences, and center logistics. Prospective randomized studies evaluating live birth rates and long-term outcomes are needed to further support clinical decision-making.

## Data Availability

The research data are described in the article presented.
