# Supplementary material for: Endometrial preparation for embryo transfer: reproductive outcomes of modified natural cycle versus hormone replacement therapy
Source: Rev Bras Ginecol Obstet. 2026 Oct 30;48:e-rbgo66. doi: 10.61622/rbgo/2026rbgo66 (PMC13754076; doi:10.61622/rbgo/2026rbgo66)
Supplement: Supplementary file 1 [file 1806-9339-rbgo-48-e-rbgo66-Suppl01.pdf]

# Supplementary Material

**Table 1S.** Multicollinearity diagnostics (VIF)

| Variable | VIF |
|---|---|
| Endometrial preparation | 1.34 |
| Age at oocyte retrieval | 1.09 |
| Infertility duration | 1.28 |
| Endometriosis | 1.46 |
| Unexplained fertility | 1.42 |
| Embryonic stage | 1.14 |
| PGT-A | 1.12 |

**Table 2S.** Distribution of baseline and clinical characteristics according to embryo development stage (Cleavage vs Blastocyst)

| Characteristic | Embryo development stage | |
|---|---|---|
| | Cleavage N = 46 n(%) | Blastocyst N = 139 n(%) |
| Anovulation | | |
| No | 42(22.7) | 116(62.7) |
| Yes | 4(2.2) | 23(12.4) |
| Genetic disease | | |
| No | 44(23.8) | 131(70.8) |
| Yes – Total | 9(5) | 1(0.5) |
| Yes –Breast Cancer | 0(0) | 5(2.7) |
| Yes – Ovarian cancer | 0(0) | 2(1.1) |
| Yes – Turner Syndrome | 2(1.1) | 2(1.1) |
| Mosaicism (45, X / 46, XX) | | |
| Endometriosis | | |
| No | 35(18.9) | 96(51.9) |
| Yes | 11(5.9) | 43(23.2) |
| Tubal factor | | |
| No | 40(21.6) | 124(67) |
| Yes | 6(3.2) | 15(8.1) |
| Advanced maternal age | | |
| No | 26(14.1) | 62(33.5) |
| Yes | 20(10.8) | 77(41.6) |
| Uterine myomatosis | | |
| No | 36(19.5) | 129(69.7) |
| Yes | 10(5.4) | 10(5.4) |
| Unexplained infertility | | |
| No | 40(21.6) | 115(62.2) |
| Yes | 6(3.2) | 24(13) |
| Male factor | | |
| No | 21(11.4) | 77(41.6) |
| Yes | 25(13.5) | 62(33.5) |
| PGT-A | | |
| No | 46(24.9) | 119(64.3) |
| Yes | 0(0) | 20(10.8) |

Data are presented as absolute and relative frequencies. No inferential statistical tests were performed for subgroup comparisons.

**Table 3S.** Descriptive statistics and normality assessment of continuous variables

| Variable | Mean | Median | SD | Q1 | Q3 | W statistic | p-value | Distribution |
|---|---|---|---|---|---|---|---|---|
| Age at oocyte retrieval | 37.60 | 38.00 | 3.48 | 35.00 | 40.00 | 0.9836883 | 3.224e-02 | Non-normal |
| Duration of infertility (years) | 4.30 | 3.00 | 2.92 | 2.00 | 6.00 | 0.8934479 | 7.575e-08 | Non-normal |
| Endometrial thickness (mm) | 8.66 | 8.30 | 1.86 | 7.50 | 10.00 | 0.9546653 | 5.805e-05 | Non-normal |
| Number of embryos transferred | 1.57 | 2.00 | 0.52 | 1.00 | 2.00 | 0.6716868 | 9.513e-19 | Non-normal |
| Number of gestational sacs | 0.43 | 0.00 | 0.59 | 0.00 | 1.00 | 0.6769891 | 1.324e-18 | Non-normal |

Shapiro–Wilk test was used to assess normality. A p-value < 0.05 was considered indicative of non-normal distribution. Variables with non-normal distribution were analyzed using nonparametric methods.
